# Summer Epidemics

**Published:** 1852-09

**Authors:** 


*Summer Epidemics.*—Formerly, physicians had considerable to say upon summer epidemics, as though they were a fixture at certain times and in certain sections of the country. It is true that there are occasional intimations of a tendency that way, and the mortality of a city increases as the summer advances; but the very general cultivation of the land in New England, the drainage of fields in the neighborhood of villages, careful attention in locating dwellings, the facilities for procuring pure water, and the increased intelligence of the inhabitants generally, as manifested in other respects, has had something to do in overcoming an epidemical tendency. In old houses, thickly clustered together, so that the sun cannot penetrate into all the crooked by-ways in which they are situated, and in which the poor and negligent usually monopolize every apartment that will give a tolerable shelter, the most formidable types of malignant disease are liable to manifest their energy. The conservators of the public health are quite familiar with this fact, and by keeping a vigilant eye on such receptacles of filth, neglect and poverty, they are often able to prevent a sudden outbreak of disease. Practitioners of medicine, too, understand better than formerly how to meet these messengers of terror.—It is reasonable to expect, therefore, that the community will not suffer, as in times past, either in imagination or reality, from sudden developments of anomalous summer distempers, coming and going without being clearly understood beyond the destruction that marked their track. —*Ibid.*

